# Wealth, household heterogeneity and livelihood diversification of Fulani pastoralists in the Kachia Grazing Reserve, northern Nigeria, during a period of social transition

**DOI:** 10.1371/journal.pone.0172866

**Published:** 2017-03-03

**Authors:** Marie J. Ducrotoy, Crawford W. Revie, Alexandra P. M. Shaw, Usman B. Musa, Wilson J. Bertu, Amahyel M. Gusi, Reuben A. Ocholi, Ayodele O. Majekodunmi, Susan C. Welburn

**Affiliations:** 1 Division of Infection and Pathway Medicine, Edinburgh Medical School: Biomedical Sciences, College of Medicine and Veterinary Medicine, The University of Edinburgh, Chancellor’s Building, 49 Little France Crescent, Edinburgh, United Kingdom; 2 Centre for Veterinary and Epidemiological Research, Department of Health Management, Atlantic Veterinary College, University of Prince Edward Island, 550 University Ave, Charlottetown, PE, Canada; 3 Avia-GIS, Risschotlei 33, Zoersel, Belgium; 4 Nigerian Institute for Trypanosomiasis Research, Ungwan Rimi G.R.A., P.M.B., Kaduna, Nigeria; 5 Brucellosis Research Unit, National Veterinary Research Institute, P.M.B 01 Vom, Plateau State, Nigeria; University of Ottawa, CANADA

## Abstract

**Background:**

A mixed methods study was undertaken in the Kachia Grazing Reserve of northern Nigeria. Surveys in March, June and October 2011 included focus group discussions, key informant and in-depth household interviews, concerning livelihood practices, animal health, ownership, and productivity. In May 2011, 249 Fulani families fleeing post-election violence entered the reserve with their livestock, increasing the number of households by one third.

**Results:**

Despite being settled within a grazing reserve, over half of households sent all their cattle away on seasonal transhumance and another third sent some away. Cattle accounted for 96% of total tropical livestock units (TLU), of which 26% were cattle kept permanently outside the reserve. While all households cited livestock as their main source of income, 90% grew crops and 55% derived income from off-farm activities. A multiple correspondence analysis showed that for each extra member of a household its TLU value increased by 2.0 [95% CI, 1.4–2.7], while for each additional marriage its TLU increased by 15.7 [95% CI, 7.1–24.3]. A strong association was also observed between small herds, small households with only one wife, alongside marked geographical wealth differences within the reserve. New immigrant families had larger household sizes (33) and livestock holdings (122 TLU) than old settlers (22 people and 67 TLU). Prior to the mass immigration, the distribution of TLU per person was unimodal: 41% of households were classified as ‘poor’ and 27% as ‘medium’, whereas post-immigration it was bi-modal, with 26% classified as ‘very poor’ and 28% as ‘medium’.

**Conclusions:**

While cattle remain the principal source of Fulani income and wealth, the inhabitants of Kachia Grazing Reserve have diversified their livelihood strategies to respond to changing circumstances and stress, especially the limited availability of grazing within the reserve and political insecurity outside, resulting in continued transhumance, the maintenance of smaller livestock holdings and pushing households into poverty.

## Introduction

The Fulani peoples are the major pastoralist group across West Africa and have dominated cattle production in Nigeria for centuries [1]. Also known as *Fulbe* pastoralists, their population in Nigeria is estimated at 15.3 million [2].

In the late 1980s Fulani were estimated to manage 90% of Nigeria’s ruminants [3]. A 1992 livestock survey found that Fulani pastoralists, the great majority of whom have now settled, grow crops and practice a form of limited seasonal transhumance, kept 83% of the cattle in Nigeria. Many arable farmers also practice animal husbandry. Traditional management in and around rural villages by non-Fulani accounted for 17% of cattle. Only 0.3% cattle were reared on commercial holdings in a peri-urban or urban settings [4]. Village and urban cattle keeping is increasing as business people invest in the current agricultural revolution in Nigeria and the local ‘indigene’ populations learn herd management skills from the Fulani. In 2014, the ruminant population of Nigeria was estimated at 19.4 million cattle, 40.6 million sheep and 71.0 million goats [5].

Traditionally, Fulani practiced year-round nomadism, partly in response to the need to migrate away from the high infection challenge presented by tsetse flies. Before the 1950s, herds from the northern savannah zone only grazed in the sub-humid zone further south during the dry season, when the risk from trypanosomiasis was lower. Since the 1950s there has been a southwards shift into the sub-humid zone for year-round grazing with Fulani pastoralists occupying 5% of the rural population of what was an inhabited zone.

By 1988 it was estimated that the dry season cattle population decreased by approximately 40% in the wet season [6] indicating that an increasingly year-round population was present in this zone. Expansion of cultivation has reduced suitable tsetse habitat, making the area more hospitable to livestock keepers [7]. An increasing number of Fulani are giving up the wet season migration northwards, to engage in mixed crop/livestock farming and a more settled lifestyle [8]. Most Fulani now have permanent homesteads and practice only short-range dry and wet season transhumance, in part due to diminishing access to rangelands from farming pressure, increasing conflicts and insecurity [1].

Grazing reserves were established in Nigeria in the 1960s to encourage pastoralist sedentarisation [9]. The reserves were anticipated to increase productivity, providing critical resources for livestock keeping (water and land tenure) and access to markets, and to reduce clashes between pastoralists and crop farmers driven by competition for resources. The Kachia Grazing Reserve (KGR) was established by the Kaduna State Ministry of Animal and Forest Resources in 1967 to settle nomads in one location to improve their standard of living; to improve the quality of livestock produced; to reduce conflict between nomads and farmers and to provide an area for research [10]. The KGR is home to some 10,000 Fulani pastoralists and their 40,000 cattle. In May 2011, a month after the presidential election, KGR experienced a sudden influx of displaced families fleeing violent clashes in their areas of origin [11].

The study objectives are two-fold. Firstly, we describe and assess variation in KGR household characteristics in terms of the household head, wives and marriages, livelihood strategies, livestock keeping, crop farming, off-farm sources of income, mutual assistance and gendered-wealth holdings. Secondly, we explore whether livestock and wealth are equally distributed among KGR households, and if not, what variables account for the variation seen across households.

The KGR, the first grazing reserve to be established in Nigeria, is representative of Fulani livelihood diversification, wealth and household heterogeneity in a grazing reserve setting. Analysing the social and economic make-up of grazing reserve communities and their resilience to social change is pertinent because of the societal and political lobby for sendentarisation of pastoralist populations. Pastoral livelihoods are in a transitional state and understanding the household economy is crucial in achieving sustainable and effective development initiatives.

### Study site

The KGR is situated in Kaduna State, north central Nigeria, and comprises 31,000 hectares between latitudes 10°03’-10°13’N and longitudes 7°55’-8°06’E. KGR lies within the sub-humid zone, 700–900 m above sea level and is fed by the Kaduna River. KGR exhibits northern Guinea Savannah woodland vegetation. The climate is tropical sub-humid, with a wet season running from June-October and dry season between November-May. The average temperature is 28°C (minimum of 19°C in January and maximum of 39°C at the start of the rains).

**Fig 1:**
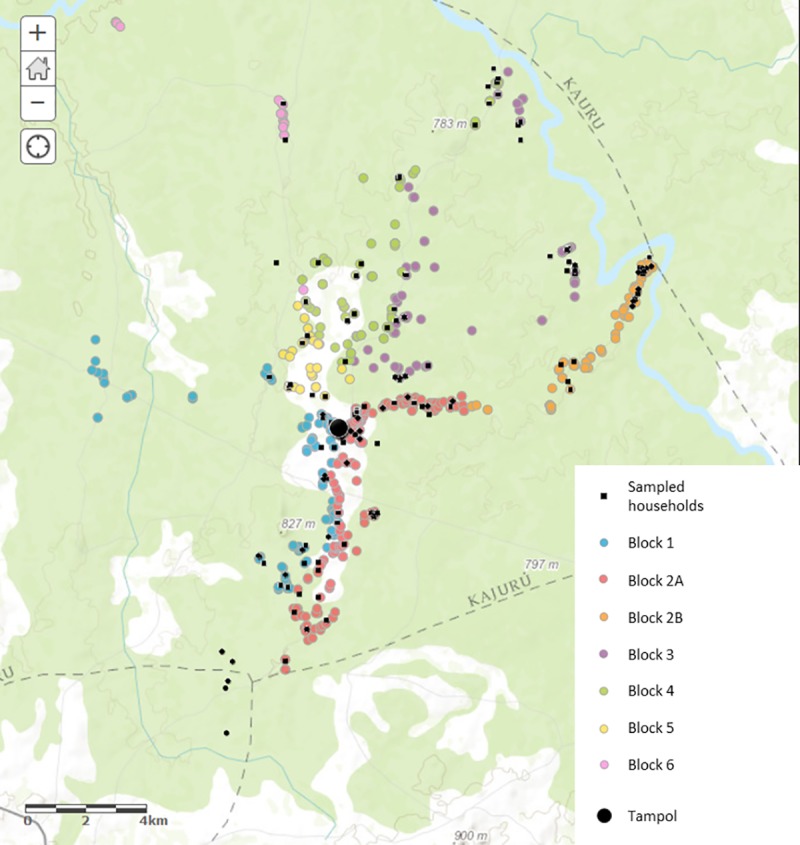
Location of KGR households. (Map was created using ArcGIS® software by Esri. ArcGIS®—with the following attribution: Esri, HERE, Garmin, USGS, METI/NASA, NGA).

KGR settlers are exclusively Fulani pastoralists. The KGR ‘district’ is called *Ladduga* or ‘bush’ in Fulfulde and the KGR headquarters and trading centre is called *Tampol* (after the tarpaulins that covered the first market stalls). Administratively, KGR is divided into 6 blocks. Block 2 is large and diverse, geographically, and is subcategorised into 2A and 2B (Fig 1). KGR has 9 *Ardos* or village heads, each representing a clan. The settlement areas within the blocks are named after the clan elder.

### Study design

This mixed methods study comprised three comprehensive livelihoods surveys undertaken within KGR during 2011: March (mid-dry season), June (beginning of wet season) and October (end of wet season).

This approach enabled themes to be covered not only through administration of questionnaires at different time points and to different cohorts of households/individuals but also through application of a range of different participatory research methods. Triangulation was employed to validate the repeatability of data obtained and ensure better reliability of evidence. This method also ensured that variations in characteristics, knowledge, perception and practices were captured.

The household was the primary unit of assessment. In July 2010, a state census undertaken by the KGR Project Office recorded 581 households in KGR. For the survey undertaken in March 2011, 64 households were randomly selected from this total. In May 2011, one month after the presidential election, KGR experienced a sudden influx of displaced families fleeing violent clashes in their areas of origin. In total, 249 families (3,000 people) moved into the reserve with their livestock (20,000 cattle, 5,000 sheep and 1,500 goats). A census undertaken in June 2011 provided a revised figure of 777 households with a human, cattle, sheep and goat population of approximately 10,000, 40,000, 10,000 and 5,000 respectively [11]. Of the 752 households for which data on the year of settlement in KGR are available, 28.2% were established in the KGR before the period of inter-communal violence that began in the early 2000s. A further 38.7% settled in KGR between 2001 and 2010 and 33.1% of all households had moved into KGR in May 2011. Of these 249 households, all were inhabiting the reserve in October 2011 intending to settle permanently. In this study, households that moved into KGR during the mass immigration event of May 2011 are referred to as ‘new immigrant’ households and the remainder as ‘old settlers’.

For each of the June and October surveys, 40 households were randomly selected from across these 777 households by allocation and generation of random numbers using the Survey Toolbox®.

#### Focus group discussions and key informant interviews

Focus group discussions [FGD] employing participatory rural appraisal techniques [12] were undertaken by the first author with 8 groups of 6–12 individuals of the same sex, with the assistance of a local translator. These discussions were supplemented by two key informant interviews. Topics of discussion and individuals/groups targeted are summarised in Table 1.

Wealth and poverty were assessed using participatory wealth ranking, in which focus group discussants self-determined wealth reference points [13].

**Table 1:** Topics and target groups and individuals for focus group discussions and key informant interviews.

Topic	Target group
Focus group discussions	
Community wealth ranking	Men and women
Role of household head	Men and women
Household revenue and livelihood diversification	Men and women
Sale of dairy products	Members of women's cooperative
Household composition, expansion and dissolution	Women
Gendered wealth holdings	Men and women
Key informant interviews	
Crop farming, KGR past and future	Elderly, educated, elite male; advisor to district head
	President of dairy cooperative
Household expansion and dissolution	Young educated community member
Grazing reserves and mobility of pastoral communities	National Livestock Development Project, Federal government

#### Questionnaires

A questionnaire was administered to each selected household. Interviews were undertaken by the first author with the assistance of a local translator. Respondents were household heads or, in a minority of cases, their sons or brothers. Not all selected households agreed to be interviewed. For the survey undertaken in June 2011 a single ‘outlier’ household was removed from analysis, having a household size of 277 and cattle herd of 1,500. Questionnaires focussed on four themes: household size and composition; the domestic animal population (species composition and holdings kept in and outside the KGR); household livelihoods strategies and sources of income. Livestock capital was used as the primary proxy for wealth. FGDs indicated that the number of animals was the most important parameter for ranking a household’s wealth status in the KGR as previously reported [14,15].

To aggregate the livestock species maintained by a household, the total number of tropical livestock units (TLU, equivalent to 250 kg live-weight) were calculated. The following conversion factors were applied: cattle = 0.70, sheep and goats = 0.10, domestic fowl/poultry = 0.01 [16]. Other wealth indicators including number of buildings, hectares farmed, and educational level of the household head, were also examined.

Our analyses are based on a total sample size of 133 households. Fifty-six households were interviewed in March 2011; 38 in June 2011 and 39 in October 2011. For the surveys undertaken in June and October 2011, approximately 30% of households were of new immigrants.

Data pre-dating the immigration event have been analysed separately and these excluded households and livestock that were on dry season transhumance. For most analyses, responses from June and October 2011 are aggregated.

#### Statistical analyses

A range of univariate analyses (t-tests, chi-square tests and simple linear regression) were carried out in R v3.1.1 [17]. A multi-variable general linear regression model to explore the key variables affecting the total livestock units within a household was created in R (regression modelling strategies ‘rms’ package) using a stepwise, forward-selection approach with Akaike information criterion (AIC) values.

Multiple correspondence analysis (MCA) was performed using selected variables from the June and October 2011 survey data. The variables used were: household size, herd size, number of marriages of household head and sources of extra income (from the options: wages (casual labour), salary (salaried work) and some sort of business initiative–e.g. tea shop, motorcycle servicing, etc.). Wealth status (using TLU per capita as a proxy) and geographical location were included as supplementary variables, which does not affect the creation of the main dimensions but enables these variables to be projected onto the MCA plot. The MCA was performed in STATA v.13 (Statacorp LP, College Station, TX, USA).

### Ethics statement

Ethical clearance for interviewing of human subjects was granted on 7th February 2011 by the Ministry of Health, Kaduna State (Nota MOH/HS/PER/VOL.I/234/70). Study participants were briefed on the purpose of the study and verbal informed consent was obtained. Written consent could not be obtained as the large majority of study participants were illiterate. Participant consent was documented directly in the questionnaires used to interview the study participants. The ethics committee of the Ministry of Health, Kaduna State, approved this consent procedure.

## Results

### Household characteristics

#### Household head

The nucleus of all KGR Fulani households is its head (HHH) or *jewuro*, an adult male, who makes decisions on social, economic and political matters. FGD responses indicated that the main role of the HHH was to manage the herd or agricultural unit, being responsible for all aspects of herd security, maintenance and reproductive efficiency.

HHH ages ranged from 23 to 87 years with a mean and median age across the surveys of 53 years. Over 50% of HHHs were aged between 45 and 64. There was a significant relationship between household size and age of the HHH (p< 0.01), though linear regression indicated that this relationship accounted for only 8% of the variation in household size.

Rates of formal education (primary, secondary or further), other than in Koranic schooling were low, at just over 10% of all HHHs.

#### Wives and marriages

A Fulani man may take a maximum of four wives at any one time in accordance with Islamic rules. It is important to consider not just the current number of wives of HHHs, but also the number of marriages contracted. Following divorce or death of a spouse, children usually remain in their father’s household.

The majority of HHHs, 70%, had either one or two wives. Two was the modal number, when all of a HHH’s marriages were considered, with 32% in the March survey and 44% in the June-October surveys having married twice. Three HHHs had married five times and one HHH interviewed had married 10 times, but was exceptional (Table 2).

**Table 2:** Number of marriages of HHH.

Category	Number of wives/marriages											
	March Interviews (n = 56 HHH)						June-October (n = 77 HHH)					
	0	1	2	3	4	>4	0	1	2	3	4	>4
Current wives	0	19	21	11	5	0	2^2^	21	32	15	7	0
Deceased wives	45	9	1	1	0	0	67	6	3	1	0	0
Divorced wives	47	5	1	2	0	1^3^	77	0	0	0	0	0
**Marriages**^**1**^	**0**	**11**	**18**	**17**	**7**	**3**^**4**^	**0**	**17**	**34**	**17**	**8**	**1**^**5**^

^1^ Number of times HHH married, inclusive of current, divorced and deceased wives

^2^ A HHH had one and another had 2 wives, but these wives died leaving both HHH with no wives

^3^ HHH divorced 5 wives

^4^ 2 HHH had 5 wives overall and 1 HHH had 10 wives (same HHH as the one who divorced 5 wives)

^5^ HHH had 5 wives, 4 present and 1 that died

#### Household composition

The household, or *wuro*, is a group of agnatically related men, their wives and children. The FGDs with women revealed two *wuro* structures in the KGR: a three-generation household in which the HHH is elderly and his sons and their wives and children live under his directive and one in which the HHH has died and is replaced by his eldest son, who lives in the same household with his junior brothers, their wives and their children. The first *wuro* structure was more common: only 11% of households interviewed reported having a HHH living with his brothers and his brother’s wives and children.

Respondents reported four phases of household expansion and division. (i) The household expands through the offspring of the HHH and his first wife, and may continue to expand to form a compound family if the HHH takes on more wives. (ii) The household expands when the sons of the HHH take their own wives and have children. (iii) Division occurs as sons and their wives separate from their father’s household if the sons have built up large enough cattle herds, as illustrated by this statement from a young focus group discussant who decided to ‘*go it alone*’ and create a new wuro distinct from the one of his father on account of his large herd size and financial independence. A son from a poor household may also be driven to leave his father’s *wuro* to improve his prospects by moving elsewhere: *‘if someone does not have enough cows to give to all his sons then he will send his son to go and work for another herd so that he can work to earn a calf*, *the going rate is two years for a female and one year for a male’*. (iv) Household dissolution occurs when the HHH dies and his herd is distributed amongst his sons and daughters in a 2:1 ratio. At this stage, each son may form his own household unit, although a household may continue to exist as a single unit even after the death of an elderly HHH. Certain factors such as death of a father, livestock wealth or poverty make household division more likely, but focus group discussions revealed that there is no typical threshold number of cattle or prescribed rule for an individual deciding to form his own household unit. The decision to divide is made by the household head, as illustrated by this statement from a focus group discussant: ‘*a son will only separate his animals and family if his father gives his approval’*.

FGDs in March 2011 showed that marriage occurs in individuals of 16 years or more. For the surveys undertaken in June/October 2011, 16 was considered the age of adulthood, accordingly 53% of the population were children (Table 3). Overall 51% of the household population was male; within the 5–15- year old age group, 60% were male. Marriage of young girls may have resulted in their being classified as older than they were. In the March 2011 survey, 16% of households reported hiring non-blood related ‘cattle boys’, classified as members of the households, accounting for 1.4% of the population. FGDs indicated these could be from non-Fulani ethnic groups.

**Table 3:** Household size and composition, June and October 2011 surveys.

Category	No HHs	Sum	Mean^1^	SD^1^	% of total population
(ages in years)					
HHH	77	77	1.0	0.0	3.9
Wives of HHH	75	158	2.1	0.9	7.9
Child < 5	71	434	6.1	4.4	21.7
Child 5–15 male	70	372	5.3	4.3	18.6
Child 5–15 female	62	253	4.1	2.7	12.7
Unmarried adult male >15	42	144	3.4	2.3	7.2
Unmarried adult female >15	35	97	2.8	2.1	4.9
Married adult males >15	55	210	3.8	2.9	10.5
Married adult females >15	57	251	4.4	2.9	12.6
***Subtotal children ≤15***	75	1,059	14.1	9.5	53.1
***Subtotal new immigrants***	25	830	33.2	22.2	41.6
***Subtotal old settlers***	52	1,166	22.4	11.4	58.4
**Total**	**77**	**1,996**	**25.9**	**16.4**	**100.0**

^1^ Mean and SD [standard deviation] apply to households (HHs) containing a particular category of individual rather than overall HHs

The mean household size was found to be higher (25.9) in June-October 2011 than in March 2011 (20.4). New immigrant households were significantly larger with a mean household size of 33.2 (mean difference of 10.8 persons, 95% confidence interval [CI] of 1.4–201) compared to the old settlers (Table 3).

#### Livelihood strategies

KGR is considered by government officials to be an ‘agro-pastoralist’ community, with the implication that 25–50% of income is derived from livestock and livestock-related activities [18]. At the time KGR was set up, it was stipulated that on settlement in KGR households should be allocated 10 hectares of land, with a proviso that 4 hectares should be dedicated to crop farming. FGD interviews showed a mismatch between the perceptions of the authorities and inhabitants.

All households engaged in livestock keeping, with 97% ranking this activity as their primary source of income or subsistence. Households reported deriving more than 50% of their income from livestock which would categorise them as pastoralists. Households also engage in other livelihood strategies (cropping, mainly for subsistence, and off-farm activities). Ninety percent of KGR households grow crops, and 96% of the crop-growers ranked this activity second in terms of contribution to overall household income. Over half of KGR households engaged in off-farm activities, and ranked this activity third in terms of its contribution to the household economy. Remittances from family members living away from home and women’s crafts also contributed to the income economy of some households, although these sources were typically ranked 3 or lower (Table 4).

**Table 4:** Householder rankings of income sources.

Activity	No HHs citing income source	% of HHs	Contribution to HH income		
			(No of HHs)		
			Rank 1^1^	Rank 2	Rank ≥3
**Livestock**	133	100.0	129	4	0
**Crop farming**	120	90.2	5	115	0
**Off farm activities**	73	54.9	0	0	73
Business	49	36.8	0	0	49
Salary	40	30.1	0	0	40
Wage	13	6.4	0	0	13
**Money from family**	45	33.8	0	0	45
**Women’s crafts**	30	22.6	0	0	30

^1^ One household (HH) gave a rank of 1 to both livestock keeping and crop farming

Data from surveys undertaken in March, June and October, 2011.

Livestock, milk and, to a lesser extent, crop sales meet the cash needs of the household. These include purchase of herbs, spices and condiments for cooking, clothes, school fees, human and veterinary drugs. Small ruminant sales cover most day-to-day cash needs whilst the sale of cattle is limited to major cash needs.

There was no significant difference in household engagement in non-livestock related activities between new immigrant and old settler households (Table 5).

**Table 5:** Household engagement in non-livestock keeping activities.

Activity engaged in	Old settlers (%)	New immigrants (%)
	n = 108	n = 25
Cropping	89.8 [82.5–94.8]	92.0 [74.0–99.0]
Business	38.9 [29.7–48.8]	28.0[12.1–49.4]
Salaried work	31.5 [22.9–41.1]	24.0 [9.4–45.1]
Wage-earning work	9.3 [4.5–16.4]	12.0 [2.6–31.2]

Upper and lower 95% CI in square brackets. Data from surveys undertaken in March, June and October 2011.

#### Livestock keeping

Livestock species kept in KGR include cattle, sheep, goats and domestic fowl (chickens, turkeys and guinea fowl). Three households kept ducks and one kept pigeons). KGR households also keep small ruminants, dogs and cats: dogs for herding cattle and cats for population control of rodents that can devastate grain reserves. Cattle accounted for 96% of the overall TLUs. The contribution of each species to the overall livestock capital in terms of TLUs is shown in Table 6.

**Table 6:** Livestock and companion animal ownership, June-October 2011.

Category	No HHs	%	SUM	MEAN	SD	TLU	% TLU
All cattle^1^	77	100.0	8,919	115.8	123.2	6,243.3	95.7
*Cattle within KGR*	*71*	*92*.*2*	*6*,*780*	*95*.*5*	*105*.*7*	*4*,*746*.*0*	*72*.*7*
*Cattle outside KGR*	*29*	*37*.*7*	*2*,*139*	*73*.*8*	*90*.*9*	*1*,*497*.*3*	*22*.*9*
Sheep^2^	63	81.8	1,567	24.9	23.0	156.7	2.4
Goats^3^	54	70.1	922	17.1	26.9	92.2	1.4
Chickens^4^	74	96.1	3,243	43.8	34.6	32.4	0.5
Guinea fowl^5^	16	20.8	113	7.1	7.3	1.1	0.0
Turkeys	7	9.1	65	9.3	13.8	0.7	0.0
Dogs^6^	41	53.2	78	1.9	1.2	NA	NA
Cats^7^	28	36.4	50	1.8	1.1	NA	NA

^1^ Cattle kept within and outside KGR

^2^ 5 HHs(households) kept sheep out of KGR, of which one had no sheep in KGR

^3^ 1 HH kept goats out of KGR (this HH had no goats in KGR)

^4^ 1 HH kept chickens both in and out of KGR

^5^ 2 HHs kept guinea fowl both in and out of KGR

^6^ 5HHs kept dogs outside of KGR, only one HH had dogs both in and out of KGR

^7^ 2 HHs kept cats outside of KGR, only one HH had cats both in and out of KGR

The survey undertaken in March 2011 indicated many sub-herds being maintained by KGR households outside of KGR. Interviews conducted in June and October 2011 differentiated between livestock kept in the reserve and outside. Approximately 40% of the households sampled in June and October 2011, maintained cattle outside of the reserve. Despite these herds being smaller than those kept within the reserve (mean herd size outside 74 as opposed to 96 inside KGR), these sub-herds accounted for 23% overall TLUs.

Some households kept goats, chickens, dogs and cats outside of the reserve, this suggests homesteads were maintained outside of the reserve as these species are not transhumant. Indeed 21% of interviewed households reported owning/hiring homesteads outside of the KGR, of whom half were old settlers. Some of the new immigrants reported that their old homesteads ‘*had burnt to ashes*’ in the post-election violence. These ‘‘secondary” homesteads were mostly within Kaduna State (Kwoi, Birnin Gwari, Anchau, Kafanchan, Kagoro, Zangon-Kataf, Kachia, Fadan Kamantan). Some interviewees reported owning property in Bauchi, Plateau and Nassawara States.

Household size was larger for new immigrants, who had correspondingly larger average TLU (Table 7). In June 2011, new immigrants and old settlers had 3.2 and 2.0 TLU per capita respectively; by October 2011, both groups had just over 2.5 TLU per capita.

**Table 7:** Household size and livestock holdings in the KGR.

	June 2011		October 2011	
Settlement status	New	Old	New	Old
Number of HHs	14	24	11	28
Mean HH population	34.6	23.0	31.3	21.9
	[21.9–47.4]	[17.6–28.5]	[15.6–46.9]	[18.0–25.9]
Mean TLU kept in KGR	111.6	44.9	77.6]	54.8
	[55.5–167.7]	[31.4–58.5]	[15.4–139.9	[26.1–83.5]

Upper and lower 95% CI in square brackets

Livestock contribute to household income and subsistence primarily through sale of cattle and small ruminants to generate cash and through the consumption of milk. Discussants reported rarely eating meat:

“*we do not have a taste for meat outside of slaughtering practiced as part of Islamic religious festivals* (Eid el Kabir and Eid el Fitr), *and even then we would rather sacrifice a sheep*”.

The economic and sociocultural value of cattle in pastoral communities ranges from prestige-making, bartering potential or currency, sources of food and labour and asset saving or insurance against disasters.

In KGR, milk from cattle is sold and or consumed. The small ruminants kept are not milk producing breeds. Among the 82% of KGR households that sell milk, half of the milk that is produced is sold, mostly within the KGR community. Some women will trek to non-Fulani villages and towns outside of KGR to sell milk and milk products. KGR inhabitants are cattle-keeping Fulani and so internal demand for purchase of milk is low. Most households take milk to the KGR central market area to sell directly to teashops. Women make *nono* (yogurt) and occasionally *wara* (cheese), sold on market days to supplement cash needs for cooking ingredients or school supplies and clothing for children. The lack of a milk market chain was described as a constraint by the community. Respondents recalled a company called ‘Milkopal’ which used to operate within the reserve, collecting milk directly from households and distributing to communities outside.

#### Crop farming

Most households interviewed grew crops mostly for household consumption (Table 4). Crop farming detail was investigated during March 2011 and data refer to the old settlers in the reserve. The modal area of land farmed was 2 hectares, although some households reported farming up to 50 hectares. Half of crop farming households (51%) sold some of the crops produced and on average reported selling 40% of their produce. Less than 20% of households reported growing crops to feed livestock.

Respondents ranked the importance of each crop grown in terms of subsistence and/or cash value. Almost all households engaged in crop farming grew maize and sorghum, which ranked as the two most important crops. Around 70% of households grew sweet potatoes and yams, while 40–55% of households grew cocoyam, soybean, beans, rice, cassava and groundnuts. Fewer than 30% of households grew millet. A few households cultivated ginger as a cash crop.

The number of hectares farmed was not correlated with the year a household moved into the reserve (Pearson coefficient = 0.028). There was a moderate positive correlation between the number of hectares farmed and household size (Pearson’s correlation coefficient = 0.396, p = 0.003) and between the number of hectares farmed and TLU per household (Pearson’s coefficient = 0.431, p = 0.001).

Households with the most livestock assets were found to farm the most crops. The number of livestock owned and household size are intrinsically linked, as the ability to look after large livestock herds also depends on the availability of manpower. There was a weak positive correlation between TLU/capita and hectares farmed (Pearson’s coefficient = 0.150, p = 0.275).

#### Off-farm income sources

Over half of KGR households have diversified their livelihoods through off-farm activities (Table 4), most citing ‘business activities’ as a source of additional income. Business activities included owning shops in Tampol, the trading centre of KGR (drug shops, teashops, a phone charging shop, a motorcycle repair shop, a general provision shop, maize grinding service and a tailor shop). Respondents also reported engagement in cattle trading or operating motorcycle taxi services. One respondent was a registered contractor of an agro-services company. One respondent had a house building and another a carpentry business. Salaried employment was also reported (Table 4). Employment included: teacher, bus driver, paramedic/health worker, computer technician, policeman/other civil service roles. Fewer households cited engagement in casual waged labour but where this was reported it consisted of building and agriculture-related activities such as weeding, ridging, planting, sowing and ploughing.

#### Mutual assistance

Approximately one third of households received money from family members who did not live within their homestead (Table 4).

#### Gendered wealth holdings

Women can inherit cattle from their father. Upon the death of a household head, his cattle are distributed in a 2:1 ratio between his sons and daughters. A focus group discussant gave an example: *‘if a HHH has 25 cows*, *1 daughter and 2 sons*, *the daughter receives 5 and each son 10 cattle*.*’* Women, however, do not hold on to this cattle wealth and will usually give these animals to her sons and husband.

Transmission of cattle wealth to the next generation is also gender biased because a father will give one female calf to a newborn son but not a daughter. All subsequent calves and herd growth will usually come from this one animal, although relatives can sometimes give young boys a calf. A 28-year old discussant reported that the pregnant cow he received from his father on his second birthday enabled him to build up a herd 10 cows, 10 bulls and 5 calves.

Focus group discussions revealed sheep are also owned and managed by men but that most goats and domestic fowl are reared and owned by women: *‘if a woman has cash needs she can sell a goat or a chicken’*.

Women are also responsible for preparing and selling milk and milk products such as *nono* (yogurt), *fura de nono* (yogurt with millet), *nebam* (butter) *wara* (cheese), *nyamri* (porridge) and *kindirmo* (buttermilk). Focus group discussions with women revealed that half of milk goes to household consumption and the other half is sold. The cash generated from milk sales is managed by the household head.

The only source of independent income for women is derived from women’s crafts. Table 4 also shows that across 23% of households, women engaged in a range of activities including metalwork (flat pans), mats, soap, food products (bean cakes for sale on market days), sewing and dressmaking. A female FGD participant elaborated:

“*this enables us (women) to get some allowance for ourselves to spend on our homes and our children*”.

### Measures of household wealth status

#### Association between KGR TLU and other household variables

The associations between KGR TLU and key household variables, were explored using linear regression models for all 133 households in the study (N = 133). An initial linear model was created with household size as primary predictor of household TLU. Household size was highly predictive for household TLU but accounted for only 28% of the variation seen across the households sampled. A series of additional variables were explored applying a stepwise, forward-selection approach using the adjusted R^2^ and AIC values shown in Table 8.

**Table 8:** Association between TLU KGR and selected variables.

Model	Variables included (predict TLU)	No	Adjusted R^2^	AIC
1	HH size	133	0.28	1,468
2	HH size and Wives^1^	133	0.35	1,457
3	HH size, Wives and Location^**2**^	133	0.39	1,455

^1^ Number of times HHH has been married including current, divorced and deceased wives

^2^ Geographical location of household in reserve as defined by Block number

**Fig 2:**
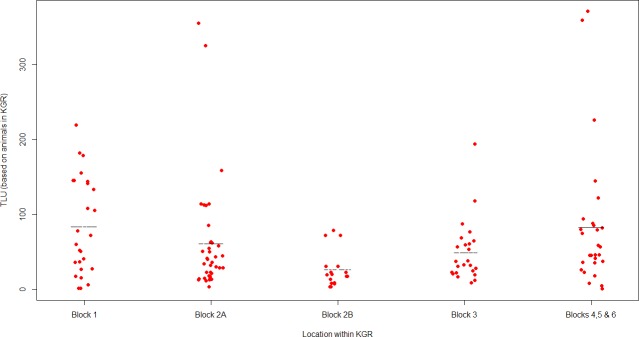
TLU values per household based on animals kept in KGR, according to the ‘block’ in which the household was located.

This analysis indicated, that in addition to household size, the total number of marriages (‘wives’) of the HHH was a significant predictor of household TLU. On average for each extra member of a household the value of its total TLU increased by 2.0 while for each additional marriage TLU increased by 15.7 (Table 9). Block membership, a variable linked to geographical location, showed marginal significance, but did not demonstrate a better fit (with a delta AIC 0.8 in the model based 121 households with complete data). A scatterplot of TLU values per household across the blocks (Fig 2) indicated differences across blocks and significant heterogeneity between households within the same block. Block 2B has the lowest median household TLU and is also the most homogeneous.

**Table 9:** Details for variables in the linear regression model of factors associated with the value of total TLU.

Variable	Coefficient	95% CI	p-value
HH Size	2.0	[1.4–2.7]	< 0.01
Wives/Marriage	15.7	[7.1–24.3]	< 0.01

Additional variables such as date of survey, old settlers versus new immigrants, years established in KGR, and number of buildings per household did not improve model fit. The introduction of various ‘off-farm’ activities: household engagement in business, salaried work and casual labour or receipt of money from family members living outside KGR also did not improve the model fit.

#### Categorisation of KGR households in terms of per capita livestock holdings

The association between wealth in terms of household TLU for livestock kept in the KGR and other key variables indicated that household size was an important variable. TLU per capita were calculated and households were allocated into wealth categories based on TLU per capita as in [19] (Table 10), an approach to the estimation of wealth status that has also been widely adopted by other authors for categorising pastoralist and agropastoralists households [20, 21].

**Table 10:** Membership of wealth categories based on KGR (KGR only livestock) and total (livestock kept in and out of KGR) TLU per capita.

Wealth category^1^	TLU/capita^1^	March 2011	June-Oct 2011	June-Oct 2011
			KGR TLU	Total TLU
Destitute	< 0.5	2	10	2
Very poor	0.-1.25	6	23	20
Poor	1.25–2.5	23	9	7
Medium	2.5–5	15	23	29
Moderately wealthy	5–10	8	11	17
Wealthy	> 10	2	1	2
**Total**		**56**	**77**	**77**

^1^ Based on Potkanski, 1997

**Fig 3:**
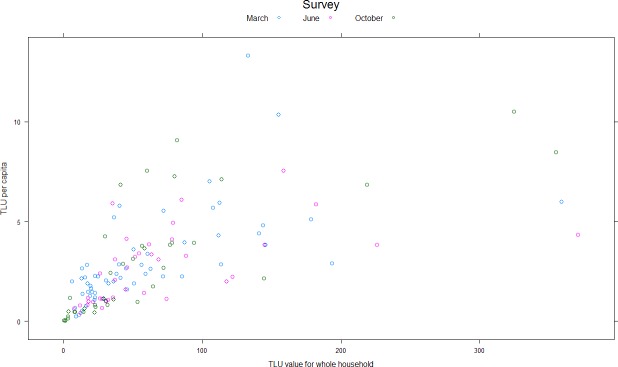
Relationship between TLU held in the KGR at household level and TLU per capita.

There was a strong relationship between per capita and overall household TLU with a linear relationship explaining around 40% of the variance (Fig 3). Introducing a quadratic term (also highly significant) improved relationship fit, increasing the total amount of variance explained by ~10%. Households with a large livestock holding tended to have large livestock holdings per person. The nature of this relationship is however, more complex than that proposed by [20] who posited a maximum ‘plateau’ at 5 TLUs per capita. Here, many households exhibited a TLU per capita higher than this value (Fig 3) and 50% of the variation in this value could not be explained in terms of overall household TLU.

A significant proportion (30%) of households in KGR change TLU-based wealth category when cattle outside of KGR are taken into consideration (Table 10). A general linear model yielded better predictions when ‘all TLU’ was considered as the outcome. Accordingly, for the MCA only data from 77 households interviewed in June and October 2011 were included in the analysis since the distinction between reported total and KGR cattle holdings was very clearly made in these interviews. Only two households fell in the lowest and highest wealth categories, these were put into the next nearest categories to generate a 4-way categorisation of wealth.

#### Association between livestock holdings and other household variables

**Fig 4:**
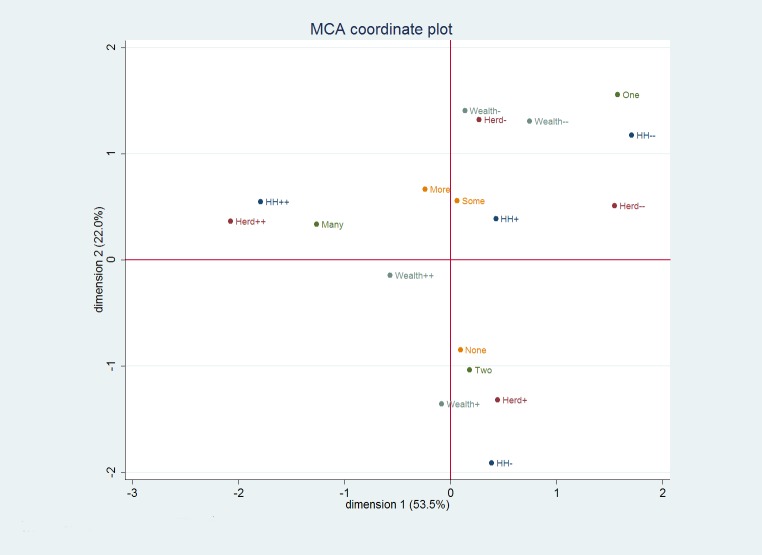
MCA coordinate plot of household characteristics.

The MCA examined the association between household wealth status in terms of livestock holdings and a range of household variables (Fig 4). Specific components associated with the creation of weights on the first two dimensions are summarised in Table 11, including household size, total TLU at household level, number of marriages and off-farm income sources. While wealth status (based on TLU per capita) is shown on Fig 4, this is a possible consequence of the fact that it was entered as a 'supplementary' variable—i.e. one that plays no part in the underlying analysis.

**Table 11:** Components associated with first and second dimensions of MCA model.

			Dimension 1		Dimension 2	
Category	Range	Inertia	Co-ord.	Contrib.	Co-ord.	Contrib.
**HH size**^**1**^						
HH—	[1–12] people	**11%**	1.707	**12%**	1.176	6%
HH-	[13–18] people	7%	0.386	1%	-1.911	**23%**
HH+	[19–30] people	3%	0.427	1%	0.389	1%
HH++	[> 30] people	**17%**	-1.789	**23%**	0.549	2%
	Overall for category	37%		38%		32%
**Herd size**						
Herd—	[1–20] TLU	6%	1.546	9%	0.509	1%
Herd-	[20–45] TLU	4%	0.27	1%	1.321	**11%**
Herd+	[45–100] TLU	6%	0.443	2%	-1.317	**16%**
Herd++	[> 100] TLU	**17%**	-2.074	**24%**	0.363	1%
	Overall for category	33%		35%		29%
**Number of marriages of HHH**						
One	Only one marriage	**13%**	1.573	**14%**	1.558	**13%**
Two	Two marriages	3%	0.179	0%	-1.037	**12%**
Many	More than two marriages	8%	-1.263	**14%**	0.338	1%
	Overall for category	24%		28%		26%
**Off farm sources of income**^**2**^						
None	None of those below	3%	0.092	0%	-0.847	**8%**
Some	[Wages, Salary, Business]	1%	0.061	0%	0.556	3%
More	More than one of above	2%	-0.239	0%	0.666	3%
	Overall for category	7%		1%		13%

Figures in bold represent the dominant elements in each category

^1^ Household size categories were selected so that the household numbers in each group were broadly similar (13 / 19 / 23 / 22 for HH—, HH-, HH+ and HH++ respectively)

^2^ Selected sources were the three which achieved the highest contribution to household rankings.

The first two dimensions of the MCA plot, accounting for around 76% of the variability due to the variables included, are shown in Fig 4. Variables used in the construction of plot are detailed in Table 11. Wealth Category was included as a supplementary variable. The first dimension is highly dependent on the largest and smallest household size categories (HH++ and HH—) as well as these two extreme categories for Herd Size (Herd—and particularly Herd++). The differences between the categories of ‘One’ and ‘Many’ wives also contribute to this first dimension. Sources of additional income make almost no contribution. The second dimension is strongly influenced by the smaller household size category (HH-) and moderately sized herds (Herd+ and Herd-). The difference between the groups having one or two wives, again has an influence, while those having no extra sources of income, separate from those with some, or many sources of extra income. The ‘wealth’ category classes are well separated particularly on the second dimension.

A cluster is observed in the top-left quadrant consisting of households which are the largest in size, have the most cattle and, also have many wives (‘Cluster 1’). Conversely, in the top-right quadrant we find households associated with very small household size, with only one wife and smaller herds (‘Cluster 2’). Finally, households with smaller overall household size but with fairly large herds and two wives are clustered towards the bottom of the graph (‘Cluster 3’). The moderately wealthy category is strongly aligned with Cluster 3, while the wealthiest class sits between all the clusters. The least wealthy households tend to align with Cluster 2. Households with smaller herd sizes and relatively large households were those most engaged in looking to sources of extra income.

Geographical location was included as a supplementary variable. Block 2B aligned closely with Cluster 2, while Blocks 4, 5 and 6 tended to align with Cluster 3 and Block 1 showed some alignment to Cluster 1. The other blocks showed no clear orientation and in general the inertia accounted for by block location was limited, indicating a fair degree of spatial heterogeneity in terms of these categories as represented within the MCA.

## Discussion

Fulani domestic units were traditionally comprised of agnatic lineages: primary kinship groups of 500–1,000 persons [22], whose common ancestor could be traced back to more than seven generations [23]. Interviews with the Fulani community in KGR show little has changed. Household members are entirely dependent on the head of household for economic, physical and moral support and for political representation in line with previous descriptions of Fulani households [23, 24]. Members of the households acknowledged that the head is responsible for management of the herding and agricultural output, for the cattle herd’s safety, maintenance and reproductive efficiency.

Within the reserve, 50% of HHHs were aged between 45 and 64 years, showing no increase from observations made almost 40 years ago [23], although older than recorded in the 1950s [25, 26]. Household demography was also little changed with 53% consisting of children, as compared to 48% reported the 1980s [23].

The average reported household size in KGR was larger than previously cited. A mean of 12 individuals was previously cited for semi-nomadic households on the Jos plateau [23] and average household size of 6 elsewhere in Nigeria [27]. In Senegalese pastoralist communities, an average household size of 11 was observed [28]. In this study, the KGR community defined a *wuro* (household) as the extended household, made up of multiple ‘*ruga*’ (homesteads), consisting of a collection of huts belonging to members of the same family. This is the unit representing a cattle-owning entity headed by the HHH even though individual cattle may in fact, belong to different family members. Differences may be attributed to interpretation of a *wuro*, as previous studies may have defined households as individual *ruga*.

In pastoralist communities, the livestock holding (particularly cattle) is considered to be the node that ties different aspects of wealth and poverty [11]. Increasing wealth is more likely to be associated with accumulating livestock than increasing cropping [29] but there is a tendency to generalise pastoralists as poor, pursuing an out-dated livelihood strategy which generates impoverishment [30]. The acquisition of stock and ensuring its wellbeing has been described as a means in itself, rather than a means to an end [31]. Although livestock, especially cattle are seen as a source of prestige, this is tightly bound up with their economic function. They are the means of production, the source of both future livestock and of daily income from milk for consumption or sale and occasional income from sale or slaughter of stock. Several authors discuss the importance of large herds for security in times of drought: ‘*a man who loses one-third of his stock is much better-off if he begins with 60 cows rather than with 6*.’ [32], a point underlined by the demographic modelling of the time taken recover from a drought event undertaken by [33]. Lastly, when a hardship strikes that is beyond remedying by the sale of smallstock, strikes, the so-called ‘unproductive’ or ‘surplus’ animals are the ones that can be sold. These different functions are reflected in cattle management practices, as observed in the KGR [34].

An increasing body of evidence describes diversification in income sources of pastoral peoples [2, 35, 36, 37, 38]. A decrease in household livestock holdings or increase in demand for household inputs is considered to favour a transition from pastoralism to agro-pastoralism, with diversification of livelihood being regarded as a risk avoidance strategy, promoting resilience to the environmental and social conditions pushing pastoral communities into poverty. A study in Kenya reported that the poorest categories of pastoralist households (those with less than 1.0 TLU per capita) had the most diversified sources of income whereas those with more than 4.5 TLU per capita focussed heavily on pastoralist activities and avoided diversification [30].

Government policy in Nigeria continues to place an emphasis on reducing Fulani mobility and promoting sedentarisation. As discussed above, although the KGR is officially regarded as agro-pastoralist, this study clearly indicates that it is predominantly a pastoralist community.

In this study, only 10% of the KGR Fulani households still relied on livestock as their sole source of income, significantly lower than the 30% observed in a recent study of Fulani households on the nearby Jos Plateau [2].

This study shows a clear association between household TLU, household size and the number of wives of the HHH. The association between family and herd size, and between prestige, polygyny and large families has been previously reported [39]. Households with more people raise more cattle, and larger cattle holdings can support more people through production of milk and cash generated by sales. While it is perceived that a cow-human equilibrium exists, at which the size of the household and herd functions as a viable unit [33], consideration of TLU/capita shows that KGR pastoralists are heterogeneous. MCA analysis revealed three main household clusters: wealthy, with large numbers of people, many wives and big herds; poor, with small household size, smaller herds and only one wife and moderately wealthy, small households, moderately sized herds and two wives.

Polygamy was regarded in male and female focus group discussants as a social marker for wealth, but marriage was described by the men as being ‘costly’, each bride coming with a ‘*bride price*’ (usually the transfer of animals from the groom’s to the bride’s family).

Taking total TLU/capita as a proxy of wealth, 38% the households interviewed in KGR in June and October 2011 would be considered destitute, very poor or poor and a further 67% showed signs of moving into poverty. KGR households are diversifying their income sources, involving other livelihood strategies and deriving income from off-farm activities including: business activities, salaried work and casual labour. For households engaged in crop farming, the number of hectares farmed is dependent on household size, as most households rely on family labour for ploughing, seeding, weeding and harvesting. Crops are grown for subsistence so that farming brings in little additional cash to the household. The extent and pattern of diversification into crop farming and off-farm sources of income varied. Households at both ends of the wealth scale were more likely to engage in off-farm income generating activities and crop farming, than those in the middle. Households with smaller herd sizes and relatively large households were those most engaged in salaried work, casual labour and business activities. Thus, households with fewer livestock had often been largely divested of their pastoral livelihood. The push out of a nomadic pastoralism and pull towards sedentarisation and diversification was eloquently described by an elderly *Ardo*:

*“there is no future in sending animals into the wilderness*. *The future for nomadic pastoralism is bleak. If we do not learn how to grow crops for our own consumption and forage, the big farmers with big farms will remain only and nomads will be boxed out of their livelihoods”*.

It is becoming increasingly difficult for Fulani men to practice transhumance in Nigeria. Younger Fulani were less sentimental towards nomadic life and more pragmatic concerning income generation. There is still prestige in having large cattle herds, but younger Fulani are open-minded about combining cattle herding with other sources of income, as one young man explained:

*“us youngsters are less motivated to have a very large herd, we are happy to get by growing crops for our families”*.

The pattern of wealth, and income distribution, among African societies dependent on animal husbandry, is one of inequality [40]. Insufficient attention has been paid to the disparities in livestock ownership and wealth differentiation [13, 41, 42, 43]. Economic inequality among pastoralists, arises from historical internal dynamics and unequal access [44]. It is important to distinguish between the distribution of livestock and wealth between households and the mechanisms which prevent permanent inequalities, such as transfer of assets and limitations on herd size imposed by family labour [45].

A geographical wealth bias was observed in KGR with one particular block of wealthier, long established settlers living near the central market and a poorer group of settlers living further away, with unfavourable access to transhumance routes and grazing reserve amenities.

At inception, the reserve was divided into 6 blocks, sprayed with insecticide and declared tsetse-free to encourage pastoralists to settle. While the division of KGR into blocks is administrative, the KGR community regard the blocks as separate and distinct entities, referring to themselves as ‘inhabitants of Block 1’ or ‘inhabitants of Block 2’. Early settlers established holdings in Blocks 1 and 2A, perceived to have the best land, the best access to transhumance routes and be best served in terms of infrastructure. Inhabitants settled according to clans and new families will settle close to relatives of the same clan. In this study, Blocks 1 and 2A contained many prosperous households and were inhabited by a large proportion of ‘first settlers’, referred to as the ‘*community elite*’. They are regarded as the wealthiest members of the KGR, with large cattle herds and many wives; considered to have supremacy over the rest of the KGR community, and exercise power by living in the most advantageous location. Most community leaders, including the District Head, the chief or representative of KGR district, live in these blocks, as described by one respondent:

*“our fellow herdsmen who have been here the longest were from wealthy clans and were able to maintain or to build up their herds better than those that came after”*.

These blocks now enjoy a prime location next to the main access road for KGR, proximity to the market, schools and other amenities (including health care), water access (boreholes and dams) and relatively large stretches of cleared woodland for crop farming. Block 2A is considered urban in character and the heart of the KGR community.

In contrast, households in Block 2B are located along a poor road leading to the Kaduna River. Households in Block 2B are regarded as the poorest members of the KGR community typically having small cattle herds. These community members live in the most inaccessible, inhospitable and remote part of the reserve, in part due to members being of a ‘poor’ clan and by virtue of their poverty. This area is furthest away from the transhumance corridor, making it difficult to take cattle out of the reserve for grazing. Proximity to the river presents a higher risk of trypanosomiasis in cattle, confirmed by a recent epidemiological survey [46]. Keeping herds close to watering points or hydrological networks has previously been identified as a risk factor for trypanosomiasis in KGR [47]. The opinions and attitudes of individuals in the community reflected these differences:

*“if we go round the Fulani settlements in the KGR we will observe that not every Fulani household is endowed with a large cattle herd, as wealth of animals is something God gives to whom he will”*.

Heterogeneity was observed between old settler and new immigrant households. Household and herd size for the new immigrants who sought sanctuary in KGR during the political clashes in May 2011, were significantly larger than observed for the old settlers. Almost all herds of over 300 cattle belonged to new immigrants. One such household owned 1,500 cattle, 80 sheep, 2,000 chickens and comprised 277 people (a TLU/capita of 3.89 placing it in a medium wealth category). The HHH had been married 5 times. The reason for new immigrants having larger herds and households was explained by KGR inhabitants as being a result of better grazing conditions in the areas in which these households had been living previously:

*“when the new immigrants fled from the violence they had to bring all their animals with them and their herds are larger than the ones we are used to here because of the abundant grasses in their places of origin as compared to the limited grazing available in KGR”*.

In response to a lack of grazing in the reserve around 40% of households also still adhered to the traditional practice of taking their KGR-based herds on transhumance at least once a year, taking cattle north during the wet season and south during the dry season travelling between 40–80 km and 20% of households seek pasture within 20–30 km of the reserve. Some 40% of KGR households also maintained permanent sub-herds outside of the reserve [11, 34].

The smaller herd sizes maintained by old settlers may be related to the level of sedentarisation. Herds that moved longer distances between seasons were much larger than herds that did not move or moved only short distances. Herd owners settled in the reserve for a shorter period had considerably larger herd sizes than those who had been in their present settlement for longer as previously reported [8]. Herd size slowly decreased over the first 10 years of settlement in the reserve, after which period herd sizes reduced sharply. By October 2011, households in the reserve reported high volumes of sales indicating that residents, in particular the new immigrants were divesting themselves of some of their animals.

In the current climate of political instability, reduced opportunities for herd mobility and poor access to pasture, many KGR residents believe that a shift from a purely pastoral to an integrated cattle rearing and crop farming system is a way in which KGR households can become more resilient. The early settlers, regarded as the elite and wealthiest members of the community owe their success to their ability to embrace crop farming: *‘when the early settlers decided to move here*, *they had to move with their dependents who did not have herds of their own*, *this stimulated them to go into crop farming as a way of reducing pressure on the needs of the household’*.

Poverty in KGR is attributed by community members to the decline of crop farming within KGR, the result of an increasing cattle population and the constraint of not having enough skilled labour: ‘*outside KGR where there are other settlers you have the privilege of hiring labour to work on your farm*. *The people in KGR are Fulani whose expertise is animals*, *not farming*, *and other tribes do not come in here’*. The solution, according to the community, is not to co-habit with other communities but for the Fulani man to develop the technological know-how and skills to grow crops for his family and forage for his animals.

The *‘Lawol-Bote’* dairy producers, a dairy cooperative in KGR, also had some very clear ideas about how the community needs to adapt to changing economic conditions. Their opinion is that fodder banking (cultivation of high yielding and drought resistant pasture crops which are then stored and fed to cattle during the dry season) is one of the ways to face the current challenges. This sentiment was shared by then current KGR Project Officer and State representative of the reserve: *‘we need the enlighten pastoralists on constant movement with animals and teach them how to make fodder banks’*.

The president of the cooperative also reported owning bulls of Friesian breed, which are crossed with the local White Fulani females to produce a crossbreed which can produce more milk whilst remaining adapted to local conditions. Shifting to a system focused on milk production through genetic breed improvement was perceived as a potential route to dairy specialisation as a way of increasing resilience, but this needs to be implemented in parallel to organised milk collection schemes and infrastructure provision (refrigerated bulk milk tank, vehicles to transport milk to milk processing plant).

The NLPD (National Livestock Development Project, Federal government) is focused on reducing the number of animals and increasing their productivity to reduce competition for limited resources and negative impacts on the ecosystem, thus it is promoting intensification and specialisation of cattle production. The federal government recognises that ‘*the state needs to do a lot more as the infrastructure of grazing reserves is decaying and cannot accommodate the increasing cattle population’*. Governmental neglect has encouraged Fulani to establish cooperatives that campaign and seek funds to promote Fulani interests.

One such cooperative in the KGR is a woman’s milk cooperative called the ‘rise of dawn’, formed to improve milk marketing opportunities within the KGR. During a FGD, its female members mentioned that a company called ‘Milkopal’ from Kaduna used to collect and purchase the milk produced by the community. Unfortunately this scheme collapsed and after years of waiting for the state to replace it, the women took their fate in their own hands and secured funds as part of the Kaduna Agricultural Development Project. This project has built a refrigerated bulk milk tank in Tampol, to improve opportunities for milk marketing. In the absence of a complementary milk collection scheme to take the milk outside of the KGR to areas where demand is high, the impact of this scheme is uncertain.

## Conclusion

While cattle remain the principal source of Fulani income and wealth in KGR, inhabitants of the reserve have diversified their livelihood strategies in response to their changing circumstances. There was a clear association between cattle holdings, number of marriages, household size, and overall wealth, with no tapering of livestock holdings per person to a threshold. A geographical wealth bias was also observed, with wealthier, long established settlers living near the central market and a poorer group of settlers living further away, with unfavourable access to transhumance routes and grazing reserve amenities.

The limited availability of grazing within the reserve and continuing political insecurity outside of the reserve are stressing pastoralist communities, resulting in the maintenance of smaller livestock holdings, pushing households into poverty and increasing diversification. Proposed strategies for further adaptation to changing economic conditions have included fodder bank schemes and shifting to a more dairy based economy (increased milk production capacity through genetic improvement of cattle) and these have been explored to some extent in the KGR. The future of the KGR’s established residents and its new immigrants will ultimately depend on their resilience and ability to adapt as already evidenced by their existing adoption of a mix of livelihood and cattle management strategies.

## Supporting information

S1 File Data set underlying the findings in this study. (XLSX)
